# Super Placebos: A Feasibility Study Combining Contextual Factors to Promote Placebo Effects

**DOI:** 10.3389/fpsyt.2021.644825

**Published:** 2021-03-04

**Authors:** Jay A. Olson, Michael Lifshitz, Amir Raz, Samuel P. L. Veissière

**Affiliations:** ^1^Department of Psychiatry, McGill University, Montreal, QC, Canada; ^2^Department of Psychology, Harvard University, Cambridge, MA, United States; ^3^Department of Anthropology, Stanford University, Palo Alto, CA, United States; ^4^Institute for Interdisciplinary Behavioral and Brain Sciences, Chapman University, Irvine, CA, United States

**Keywords:** placebo effects, contextual factors, differential placebo effects, feasibility, pilot

## Abstract

**Background:** Ample evidence demonstrates that placebo effects are modulated by contextual factors. Few interventions, however, attempt to combine a broad range of these factors. Here, we explore the therapeutic power of placebos by leveraging factors including social proof, positive suggestion, and social learning. This study aimed to test the feasibility of an elaborate “super placebo” intervention to reduce symptoms of various disorders in a pediatric population.

**Methods:** In a single-arm qualitative study, participants entered an inactive MRI scanner which they were told could help their brain heal itself through the power of suggestion. The sample included 11 children (6–13 years old) diagnosed with disorders known to be receptive to placebos and suggestion (Attention Deficit Hyperactivity Disorder, Tourette Syndrome, chronic skin picking, and migraines). The children were given positive suggestions during 2–4 placebo machine sessions over the span of approximately 1 month. We assessed open-ended treatment outcomes via recorded interviews and home visits.

**Results:** The procedure was feasible and no adverse events occurred. Ten of the 11 parents reported improvements in their children after the intervention, ranging from minor transient changes to long-term reductions in subjective and objective symptoms (e.g., migraines and skin lesions).

**Discussion:** These preliminary findings demonstrate the feasibility and promise of combining a broad range of contextual factors in placebo studies. Future research is needed to assess the causal effects of such interventions.

## 1. Introduction

Many elaborate medical procedures are essentially placebos. Some surgical interventions for osteoarthritis (1) or Parkinson's disease (2) do not outperform sham versions of the procedures, nor do some neurological interventions such as brain stimulation for depression (3, 4) or EEG neurofeedback for ADHD (5, 6). To be clear, these procedures may still be effective, but placebo factors play a substantial role. These strong placebo effects may be partly due to the perceived complexity of the intervention. Some evidence suggests that sham procedures or medical devices that appear more complex have stronger effects (7–9); for example, sham surgeries and placebo acupuncture are generally more effective than inert pills (10). Beyond perceived complexity, numerous contextual factors modulate placebo effectiveness, including the perceived cost of the procedure (11, 12), colour of the pill (13), presence of other patients (14, 15), competence and warmth of the healthcare provider (16–18), and expectations of the patient (19). Given the importance of these contextual factors, some researchers argue that placebo effects should be reconceptualised as *contextual healing* with more emphasis on these various performative elements (8). Thus, placebo researcher Ted Kaptchuk (8) states that “with good showmanship, a well-designed, totally inert stage prop … can produce exaggerated placebo effects.” As an initial test of this hypothesis, the present study combined various contextual factors in an attempt to promote placebo effects, involving a “showman” (a celebrity science communicator), a film crew, and an elaborate (but inactive) MRI scanner serving as a placebo machine. Our aim was to assess the feasibility of an intentionally elaborate intervention uniting insights from placebo science with the allure of cutting-edge neuroscience technology.

Although researchers have identified various contextual factors that modulate placebo effects (8, 20, 21), relatively little research tests how these factors may be combined. It is unknown whether the majority of these factors can be combined to produce additive placebo effects (22). Instead, some factors may be redundant in the presence of others; for example, the perceived cost of a placebo procedure may be irrelevant if the procedure appears sufficiently complex, as in surgical or neurotechnological interventions. Other combinations of factors may even suppress effects, for example if patient expectations become unrealistically high and lead to a loss of confidence following only modest improvements (19).

There are two strategies for testing combinations of contextual factors in placebo studies. Bottom-up studies test a small number of factors to determine the most effective combination. For example, Howe et al. (16) tested the effects of a placebo cream in a design crossing the healthcare provider's perceived competence and interpersonal warmth; placebo effects were strongest when the provider exhibited both. Kaptchuk et al. (23) compared a wait-list control group, a sham acupuncture intervention, and an “augmented” intervention with more practitioner attention and confidence; placebo effects increased with each step. Given the large number of contextual factors and possible combinations, bottom-up studies proceed slowly but with high experimental control.

Top-down studies take the opposite approach. Here, researchers use a “kitchen sink” strategy by combining numerous factors at once. If placebo effects are strong, subsequent studies can dismantle the effects by hypothesising the important factors or combinations. For researchers developing interventions, top-down studies can rapidly test whether a particular combination of factors is feasible and potentially effective, which can be clinically relevant even if the mechanism is unknown. Due to their complexity and lower explanatory power, top-down studies are more rare. In one study, we combined numerous contextual factors in an attempt to promote placebo effects in the context of psychedelic drugs. Using an elaborate physical environment, confederates acting out the effects of the supposed drug, and careful expectation management, we demonstrated some of the strongest placebo effects on consciousness in the literature on psychedelic drugs (24).

The control groups of brain-based intervention trials may resemble top-down placebo studies. For example, the transcranial magnetic stimulation procedure contains “almost all conceivable factors that might enhance placebo effects,” including complex scientific machinery, medical paraphernalia, interactions with experts, a credible institution, and plentiful media attention (25). We propose that mock neuroscience equipment, such as defunct MRI scanners, may serve as similarly potent placebos (26). Under the right circumstances, people can be convinced that neuroscience equipment can read their mind (27), insert thoughts into their head (28, 29), influence their task performance (30), move their limbs (31), or even evoke mystical experiences (32). In an earlier top-down study, we gave participants verbal suggestions that an elaborate (sham) brain scanner could activate brain areas to insert thoughts into their head. Not only did most of the participants believe this, many also reported unusual experiences inside the scanner, including headaches, involuntary movements, psychosomatic sensations, and reduced feelings of control (29). We suspected that a similar intervention could be adapted to the clinical domain.

Accordingly, we developed an elaborate intervention which leverages the cues, props, and rituals of the therapeutic encounter as well as the cultural prestige of neuroscience equipment. We combined as many contextual factors as possible, resulting in perhaps the most elaborate intentionally placebo-based intervention in the literature. In this study, we aimed to assess the feasibility of this intervention.

## 2. Materials and Methods

### 2.1. Participants

We recruited children, since they are known to be particularly susceptible to placebo effects (33). We initially targeted children with Attention Deficit Hyperactivity Disorder (ADHD; *n*=8) and Tourette Syndrome (TS; *n*=4, often co-morbid with ADHD), two disorders known to be receptive to placebos and positive suggestions (34, 35). During recruitment we also accepted participants who contacted us with chronic skin picking (*n*=1) and migraines (*n*=1). Families were recruited through social media and mailing lists at schools for a study described as investigating a non-drug intervention based on positive thinking. We recruited as many participants as we could during the 2-month study period; in total, we recruited 11 participants aged 6–13. Parents provided informed consent and children provided assent during the first session. The procedure was approved by the McGill University Research Ethics Board III (#32-0617).

### 2.2. Procedure

We used a single-arm design, with an initial meeting followed by two to four intervention sessions depending on the severity of symptoms.

#### 2.2.1. Phone Interview

In the initial phone interview, parents described their child's symptoms and history. The parents often mentioned that they saw limited results from standard treatments or that they did not wish to put their children on medication due to side effects. We fully briefed parents on the procedure, explaining that it was non-invasive and based on the placebo effect as well as positive suggestion. Any healing would come from the child's own brain rather than from the machine. We told the parents to share as many details with their child as they thought would be helpful, but we suggested not to describe the procedure in the negative terms that are sometimes associated with placebos (e.g., as a fake or sham procedure).

#### 2.2.2. Video

We asked the parents to show their child a demonstration video featuring YouTube celebrities describing the procedure. We sent parents two videos, each including either two female (Merrell Twins) or two male speakers (father and son from *What's Inside?*). Using a script we wrote, the speakers stated that the machine helped children reduce their symptoms as well as to “relax, focus, and be more confident” by helping the brain heal itself. The video was intended to build positive expectations by leveraging principles of persuasion: celebrity endorsement and social proof (36). The speakers in the video also mentioned the uniqueness and scarcity of the procedure to suggest its value (e.g., “Not too many people get the chance to have this awesome experience”) (36). The video then showed a previous child participant to demonstrate the kinds of improvements to expect, in order to promote placebo effects through social learning (14).

We collaborated with a video production team who provided the endorsement videos, filmed the procedure and home visits, and provided a camera crew and celebrity science communicator. This (admittedly atypical) collaboration gave us the resources to maximise the persuasiveness of the procedure.

#### 2.2.3. Session 1: Interview

##### 2.2.3.1. Briefing

Approximately 1 month after the phone interview, the children and parents came to the lobby of the Montreal Neurological Institute. An assistant wearing a white coat greeted the family, built rapport, answered questions, and then led the family to a waiting area outside the brain imaging center. After a 5-min wait to build anticipation, the family entered the MRI control room to meet the experimenters (co-authors JO and SV) and a science communicator (Michael Stevens), all dressed in lab coats to increase credibility (37). Three camera operators were also present. As in our previous study (29), the room resembled that of a cutting-edge neuroscience experiment, with scientific-looking equipment and computer screens displaying brain scans (38).

After warmly greeting the families and building rapport (which can enhance placebo effects) (16), we explained to the children that we were not medical doctors but were instead scientists who study the mind. We explained that the camera crew had flown in from Los Angeles to film our novel procedure. We then asked the children if they had watched the introduction video (all but one had) and if they had any questions. Using similar language as in the video, we gave an analogy to illustrate our approach:

Experimenter: You know when you're playing outside and you get a scratch on your hand? What happens to it?

Participant: It heals.

E: And what do you have to do to make it heal?

P: It just heals on its own.

E: That's right. The body heals on its own—you don't have to do anything. That's what we study. Just as your body knows how to heal itself, your brain knows how to heal itself as well.

We then explained that the procedure involved entering “what some kids call the healing machine.” We told them the procedure works through suggestion, which we described as a “very powerful thought that can help you heal yourself.” We stated that the entire intervention—“everything that we say and do, everything you see around us, this equipment, these lab coats, as well as the machine”—is part of the suggestion procedure.

To further promote social learning and placebo effects (14), one of our pilot participants—an 8-year-old child previously depicted in the video—was present as a peer mentor. He explained that the procedure did not hurt and instead helped him reduce his migraines, which he also felt improved his concentration and confidence at school.

##### 2.2.3.2. Interview and Reframing

We then proceeded with a 15-min interview aiming to help participants focus on their strengths and build positive expectations. Rather than discussing symptoms or diagnoses with the parents, we focused on the children and asked them about their talents, interests, and what they would like to improve. We used cognitive reframing to reappraise their symptoms as latent strengths. For example, if participants described their behaviour in negative terms (e.g., hyperactive), we reframed this in positive terms (e.g., “It's great that you have all of that energy that you can use to help yourself focus on different things”).

When participants mentioned symptoms, we focused their attention on the times that the symptoms did not occur (i.e., attention to variability) (39, 40). For example, if they mentioned being hyperactive, we asked them when they felt most relaxed. We then told them, for example, “Your brain already knows how to relax itself, and you do it every time you read a book you like.” One child mentioned that he had migraines; we replied, “You already know how to *not* have migraines. In fact, 99% of your life you don't have migraines—you're already doing great at this. Let's see if we can make that 99% even higher.”

**Figure 1:**
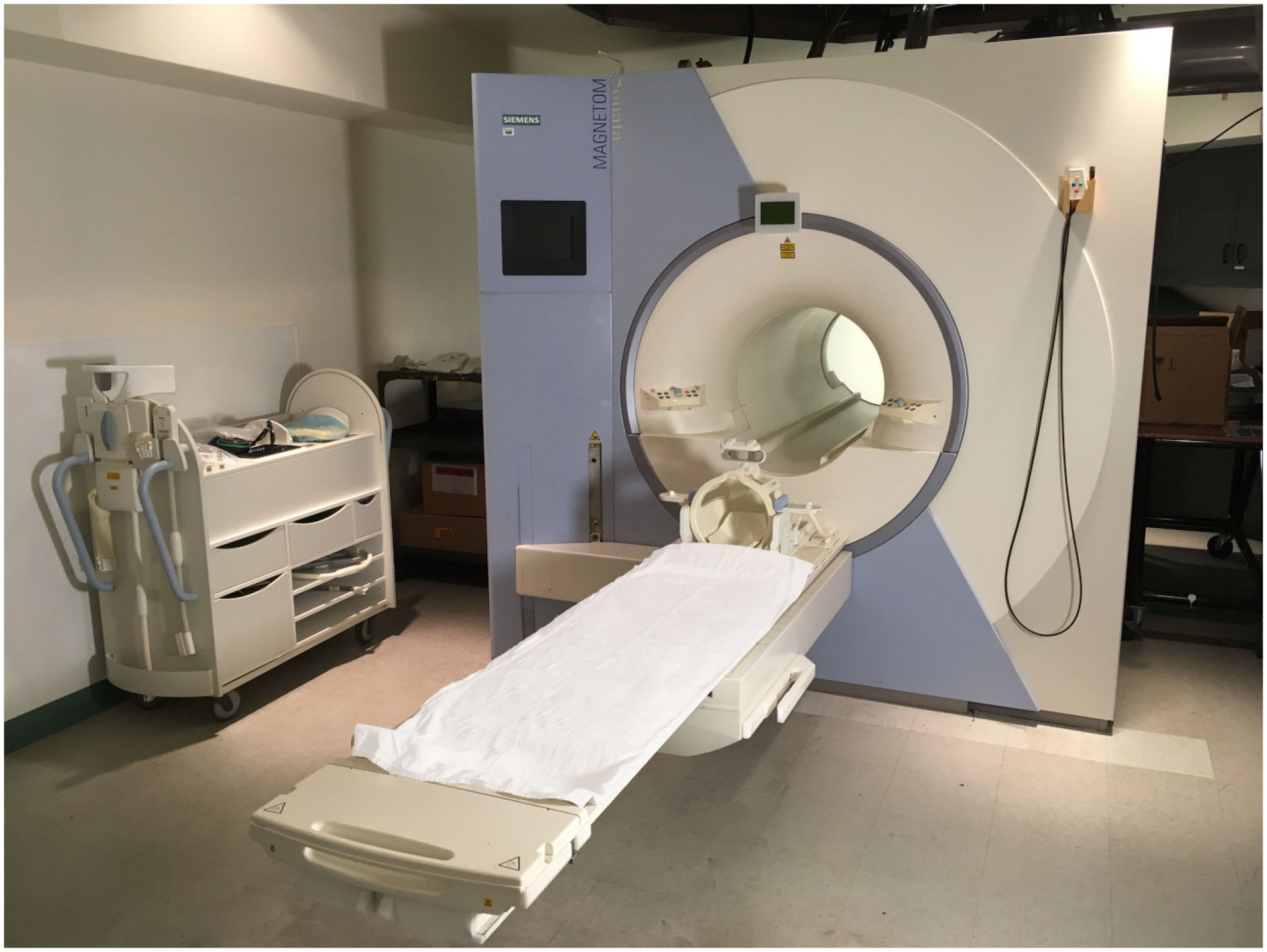
Decommissioned MRI scanner. The scanner appeared to function but the magnet was off.

We then brought families into the adjacent scanner room containing a decommissioned 1.5 T Siemens MRI scanner (Figure 1). The machine appeared to be fully functional, with lights, fans, a display screen, and a sliding table. Speakers inside the scanner played pre-recorded MRI sounds on top of mystical-sounding “space music” (*Floating Galaxies* by Nebula). We told the children that it was a “modified brain scanner that originally was used to take pictures of the brain but now helps children heal themselves through the power of suggestion.”

Participants watched the peer mentor enter and exit the scanner. He reassured participants that the procedure was safe and relaxing. We told the families to return for another session if the child demonstrated interest in entering the scanner. All 11 families returned for the next session.

#### 2.2.4. Session 2: Placebo Machine

The participants returned 1 or 2 weeks later, to accommodate their schedules. We asked them what kind of “mental superpower” they would like to improve and then framed their response as relevant to their symptoms. One participant with migraines said that he wanted to improve his memory and remove his headaches; we told him the procedure could help his brain to heal itself faster which may improve other brain functions. We then asked the children what activities they found most relaxing, in order to tailor the upcoming verbal suggestions.

Families were asked to remove any metal objects from their pockets before entering the scanner room. On the scanning table, we led the participants through a brief relaxation procedure in which they tensed and relaxed their shoulder muscles and took deep breaths. Based on their previous responses, we told them the machine would make them feel relaxed, as they do when reading a good book, for example. We asked them, “How deep would you like to go into the scanner, into relaxation, today?” To reduce claustrophobia, we let them verbally control how far they entered the scanner.

One of the two experimenters then stepped aside and quietly discussed the procedure with the parents, reminding them that the machine was a placebo. We instructed the parents to reinforce any positive changes seen in their child following these sessions and to remind them that these changes were from their child's own healing rather than from the machine itself.

The other experimenter continued to give the child suggestions that the machine would help the brain to become more focused, attentive, and calm, tailoring the suggestions to the child's desired “mental superpower.” We used direct suggestions: “As you slide deeper into the machine, you will find yourself feeling more and more relaxed and focused” (41). The brain scanner procedure took approximately 15 min.

Following the procedure, we brought the families back to the control room. We told them that although the procedure may help improve their desired outcomes, “we are more interested in what unexpected positive changes [they] will notice in the coming days and weeks.” We introduced this “surprise improvement” suggestion to promote a positive mindset and have children focus on broad and open-ended improvements. This focus would also help children confirm that the procedure worked for them, which may further increase placebo effects (19, 42).

#### 2.2.5. Session 3: Watch

One to two weeks later, the families returned to the lab. We asked them about any changes they had noticed, reminding them that these were due to the brain's own ability to heal itself and that these improvements would vary in speed. The children again entered the machine. This time, we told them we would put the machine on a higher level that would help them relax even further; the machine produced louder buzzing this session.

To help condition the placebo effects, we gave the children a watch (Octopus, Joy Family Tech, Annecy, France) to “bring part of the machine” home with them. It would buzz periodically throughout the day and show positive icons (e.g., a smile or heart); we told them each time they would feel a buzz, their brain would continue to heal itself.

#### 2.2.6. Session 4: Follow-Up

Another 1–2 weeks later, families returned to the lab for a follow-up. We interviewed the children and parents together in a 30-min semi-structured interview asking about changes in symptoms, strengths, and any unexpected effects. This formed our open-ended outcome measure. The camera crew later filmed home visits with a subset of the children showing the strongest effects. The audio from the videos and interviews was later transcribed. In two of the cases (described below), we attempted to conduct additional follow-ups to assess the longevity of the improvements. In sum, this elaborate procedure leveraged various contextual factors to promote placebo effects (see Table 1) (25).

**Table 1:** Contextual and psychosocial factors used to boost persuasion and placebo effects.

**Category**	**Factor**	**Implementation**
Equipment (7, 10, 37, 43–45)	Elaborate intervention	Large, loud brain scanner with lights and music
	Trust in neuroscience	Scientific equipment, brain scans on monitors
Learning (14, 15, 46)	Reinforcement	Parents reinforced improvements, buzzing watch
	Social modelling	Peer mentor described effects
	Conditioning	Medical paraphernalia and rituals, buzzing watch
Persuasion (36, 42)	Celebrity endorsement	Demonstration video, science communicator
	Credibility cues	Institute, affiliation logos, lab coats, identification badges, medical paraphernalia
	Scarcity	Video said intervention is open to few participants
	Social proof	Camera crew, peer mentor, research assistants
Suggestion (21, 47)	Cognitive reframing	Reframed symptoms as latent strengths, focused on when symptoms were absent
	Mindset	“Surprise improvement” suggestion, “mental superpower” focus
	Positive and hopeful expectations	Video, peer mentor, and experimenters all described benefits
	Positive suggestion	Healing suggestions, skin healing metaphor
	Relaxation	Music from machine, deep breathing, muscle relaxation, relaxing verbal suggestions
Therapeutic relationship (8, 17, 48)	Interpersonal warmth	Built rapport with participants

## 3. Results

The procedure was feasible and no unexpected issues arose. No children or parents reported any adverse effects from the procedure, beyond temporary drowsiness after the machine sessions. Overall, ten of the eleven parents reported improvements in their children following the sessions. Two children showed near-complete cessation of symptoms. We describe the (pseudonymous) participants' improvements, approximately ordered by strength, noting that the cause of these changes cannot be determined in our feasibility study.

Maria (age 12; diagnosed with chronic skin picking) had compulsively picked the skin on her arms and face for 2 years—including in her sleep—resulting in frequent skin infections. She also reported anxiety associated with showing her infected skin in public; her mother would bandage Maria's arms and face every morning. Maria was no longer comfortable doing public activities that she previously enjoyed, such as going to the swimming pool. After two sessions in the scanner, her skin picking decreased, including when she slept. We also gave her an additional session in the scanner, telling her that her skin would heal faster and faster and that her hands would no longer want to pick her skin. After the sessions, Maria reported:

At first I was confused, because I was just going into the machine and I was like, “What is this doing?” … And then after another two sessions, I started to notice you feel more relaxed, calm, confident. And I noticed I wasn't picking as often. I didn't have the urge to pick.

**Figure 2:**
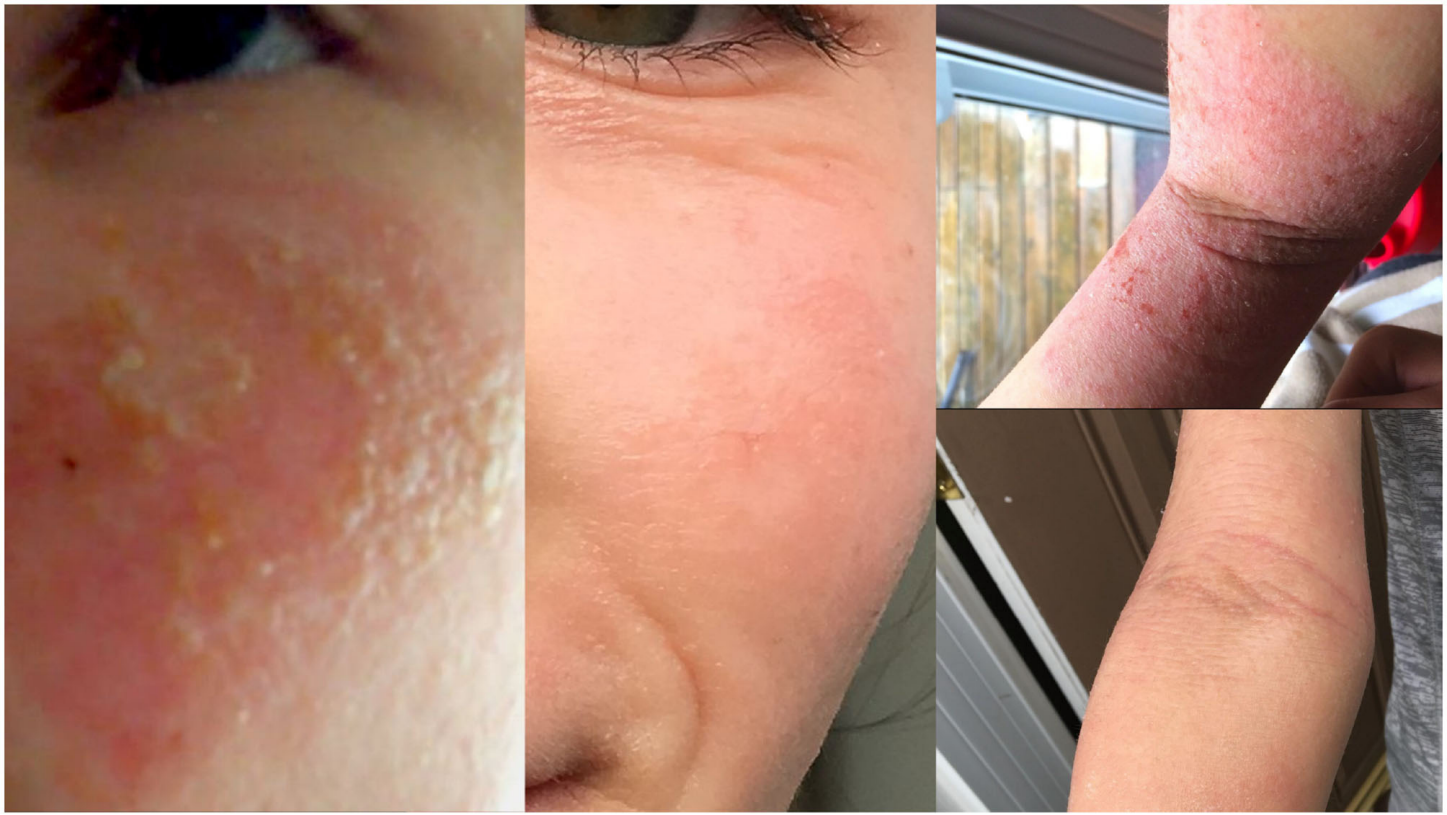
Chronic skin picking, before and after the intervention.

Her symptoms reduced to near remission (Figure 2). Her mother reported that Maria's skin “has remained intact, well-hydrated, and free from scaling and for the most part from itching as well.” Further, Maria is “laughing more, has more energy, is sleeping much better [and is] so much more calm and less anxious.” At a one-year follow-up, Maria's family reported that she no longer picks her skin. Maria also stated that she no longer needs the machine:

When you [exit the machine], you learn how to lie down and go into that same state that you were in inside the machine, and after a few sessions, you don't even need the machine any more. So if I have another problem, I can just do it myself now.

At a final 2-year follow-up, Maria continues to show no symptoms.

Nate (age 13; migraines), had daily migraines following a series of concussions which interfered with his concentration and performance at school. Nate's mother reported that their neurologist did not know why his symptoms persisted. After his first session in the scanner, he and his mother reported that his migraines had stopped. He also reported improvements in confidence, concentration, and memory. After the study, we were unable to reach the family for continued follow-ups, so we could not assess the duration of the improvements.

Ned (age 9; ADHD, anxiety, apraxia) was very relaxed after his first session and nearly fell asleep on his mother's shoulder during debriefing. According to his mother, that day he “[went] home and slept for 4 h. … He has never been able to sleep during the day; even as a baby this was extremely difficult for him to do. He was so calm and in control.” Ned had been on medication for 4 years; his mother reported that after the intervention he did not need his medication for the summer and was better able to control himself. “When he got upset, he would ask me to breathe with him; when he was anxious about something, he was able to identify what he was anxious about and talk it out with me or with himself.” His mother reported that for her family, the sessions “made a big difference in [their] quality of life.”

The rest of the children showed comparatively small effects. Vincent (age 8; ADHD and TS) had pronounced verbal and behavioural tics, and he would sometimes rub his fingers obsessively on jagged edges, causing injury. At the follow-up, his mother reported that his tics had reduced; he still sometimes reverts to rubbing his fingers but now controls on which surfaces he does so. Further, he is reportedly “much more confident, able to be alone for a prolonged period without getting anxious, and is more open to new things.”

Ishmael (age 7; Autism Spectrum Disorder and ADHD) had marked difficulties concentrating. His father reported various improvements, such as Ishmael's willingness to shower without his caretaker present, and his ability to attend summer camp and socialise with peers by himself. His teachers report (according to his father) that he seems calmer.

James (age 12; TS and ADHD), according to his father, was “a lot calmer and said that the machine helped him. … He was quite ‘zen’. He hasn't been violent; his ticks were a lot better. … It was a different James—it was like having a new child.” These effects lasted a few days after the first session and similar but weaker effects persisted for 2 weeks after the second session.

Matthew (age 12; TS and ADHD) showed improvements in his self-regulation and anger management following each session, according to his mother. Matthew said he was impressed by “the power of the machine.”

Andrew (age 11; TS and ADHD) seemed much calmer in the week following the first session, according to his father. After the second session, Andrew was reportedly “much nicer” and made a new friend, which was rare and “a big deal for him.”

Two children showed only minor improvements that were difficult to interpret. Faris (age 7; ADHD and Autism Spectrum Disorder) improved self-regulation and anger management after the sessions. These changes, however, were confounded by his starting a new stimulant medication 1 week before the first session.

Kelsey (age 13; ADHD) reported improvements in creativity and focus after the first session and stronger effects after the second. Her mother still reported Kelsey's hyperactivity. Conflicting goals and observations between mother and daughter made these improvements less clear.

Finally, one child demonstrated no noticeable improvement. Jin (age 6; undiagnosed, unspecified oppositional behaviour) was oppositional with his mother and the experimenters. He showed little interest in the procedure and expressed scepticism about the machine. One week after receiving the watch, his mother reported that he was “just a little bit calmer.” We did not notice any changes in him throughout the sessions.

## 4. Discussion

We introduced children to an elaborate placebo intervention that we told them could reduce symptoms of their disorders (ADHD, Tourette Syndrome, chronic skin picking, or migraines). There were no adverse events and ten of the eleven participants reported various improvements. One participant remains symptom-free two years later. These promising results demonstrate the feasibility of the procedure.

This feasibility study had clear limitations. Given the design and small heterogeneous sample, we cannot make any causal claims or speculate about the generalisability of the results. Further, some of the parents' reports were likely biased by self-selection, Hawthorne effects, demand characteristics, and a desire to present well for the scientists and film crew. These biases may be less likely to explain the more easily measurable effects such as skin healing and migraine cessation, but our study was intended to test feasibility rather than effectiveness. If these improvements do represent causal effects, these results would be consistent with other studies showing positive effects in the placebo control groups of neurological interventions (3–6, 49). Future studies could use standardised measures to assess improvements, such as the PedMIDAS for migraines (50) or the Test of Variables of Attention for ADHD (51).

Another limitation with our procedure is the use of deception. Schwab (52) makes the distinction between lying (knowingly saying something false) and deception (saying true statements to produce a false belief). We propose a subset of this deception could be called *implicit*, wherein the experimenter's non-verbal behaviour or the experimental context leads participants to hold false beliefs. Such subtle methods often lead to more effective deception than lying, since assumptions may be harder to question than explicit statements (53). On one hand, in our study there was little lying: we told the families that the procedure was all a performance to promote positive effects, and that any improvements would be caused by the child's own brain due to positive suggestion and expectation rather than the machine. On the other hand, the procedure used copious implicit deception; after informing families about the nature of the procedure we continued the performance as if the machine were real and powerful. Further, the machine looked and sounded like a functioning scanner. It is possible that this performance overpowered our explicit true statements about the machine (54). Analogously, several studies have demonstrated that telling audiences that a performer is a magician does not stop them from believing the magician has supernatural powers (55–57). Several of the parents unexpectedly seemed to forget about the inactive nature of the machine between the sessions, even though they showed clear understanding of the placebo component earlier. For example, several asked to see pictures of their child's brain or asked whether they could bring their phone or wallet into the machine room given the ostensible magnet.

This unexpected finding highlights the complexity of deception in placebo research, which is not often discussed in the literature (58, 59). Placebo studies tend to focus on lying and overlook implicit deception. This issue is particularly important in *open-label placebo* research, in which participants are informed that the treatment is a placebo. Although these studies rarely involve lying, there may be implicit deception (59). Researchers give open-label placebo pills in a standard prescription bottle and handle them carefully rather than with the nonchalance of, say, giving breath mints. Just as audiences can know a magicians' tricks are sleight of hand but still believe them to be supernatural feats, participants may know that they are consuming a placebo yet still somehow believe otherwise and act accordingly. Indeed, participants in open-label studies sometimes report believing the placebo is an active medication despite being told otherwise (59, 60). In this way, it may be less relevant whether researchers use implicit deception or lying if the end result is similar. Open-label placebo researchers could perhaps conceptualise deception based on its outcome (i.e., participants holding false beliefs) rather than its process (i.e., the type of deception used).

Another potential ethical issue concerns the possibility of *nocebo effects*, negative effects or worsening of symptoms following an inactive procedure. Although we saw no evidence of them here, nocebo effects are possible from technological procedures. For example, in a similarly elaborate procedure intended to produce nocebo-like effects, participants inside a sham brain scanner reported various unusual experiences, such as heat, pulsations, and a loss of control over their thoughts (29). Outside of lab environments, people sometimes report nocebo effects from electromagnetic fields (61) and wind turbines (62). When artificially increasing the perceived complexity of placebo procedures, or when using inherently complex procedures such as brain stimulation (25), researchers should take care to minimise potential nocebo effects (63, 64).

Future research could assess the causal effects of our procedure. One potential design could test the role of expectation by comparing effects when participants are told the machine is merely scanning their brain versus helping improve their symptoms. We would predict that the positive expectation may “activate” many of the contextual factors tested here. For example, the perceived complexity of the procedure may be irrelevant if participants believe it is only scanning their brain; presumably few patients experience symptom relief from an MRI scan alone. Alternatively, studies could replace the machine component with an inert pill, to compare simple and complex interventions while holding the other contextual factors constant (7, 25).

## 5. Conclusion

The more general goal of our research is to better understand how factors can be combined to promote placebo effects and reduce nocebo effects. Our intervention as tested is unlikely to be clinically feasible, given the elaborate combination of factors—including celebrity endorsements, a camera crew, various experimenters in lab coats, expensive scientific equipment, and a peer mentor. However, many of these factors may have been redundant (22); dismantling the effects could create a more minimal and feasible intervention, at least similar to other sham brain stimulation procedures. Such procedures, with the right framing, may be helpful to promote the mindset that patients control some of their improvement—that the procedure merely helps the brain heal itself. As one of our participants claimed a year later, she no longer needs the machine since she can get into the same “state” and reduce symptoms herself. This mindset may be more empowering than relying on long-term treatments, placebo or otherwise.

Understanding which factors promote placebo effects could allow these factors to be carefully leveraged in even non-placebo treatments (21, 65). For example, if social modelling and peer mentors are effective, a feasible alternative would be to have clinicians direct patients to a video showing previous patients describing their improvements (24). Currently, many of the factors involved in medical interventions are haphazard and not optimised in clinical settings (66). Training practitioners to use positive suggestions can improve treatment outcomes (67, 68); placebo-based training focusing on optimising contextual factors could be similarly effective. We hope that our promising results motivate more top-down studies to better understand the complex performative aspects of medicine and their effect on the body's healing processes.

## Data Availability Statement

The original contributions presented in the study are included in the article/supplementary material, further inquiries can be directed to the corresponding author/s.

## Ethics Statement

The studies involving human participants were reviewed and approved by the McGill University Research Ethics Board III (#32-0617). Written informed consent to participate in this study was provided by the participants' legal guardian/next of kin. Written informed consent was obtained from the minor(s)' legal guardian/next of kin for the publication of any potentially identifiable images or data included in this article.

## Author Contributions

JO and SV contributed equally to the study design, data collection, and writing. ML and AR assisted with manuscript writing. All authors contributed to the article and approved the submitted version.

## Conflict of Interest

SV has worked as a scientific consultant for the funder, Intellectual Property Corporation. He also later obtained an industry-university Mitacs grant with Joy Family Tech who provided the watches used in the study. The remaining authors declare that the research was conducted in the absence of any commercial or financial relationships that could be construed as a potential conflict of interest.
